# Does race impact speech perception? An account of accented speech in two different multilingual locales

**DOI:** 10.1186/s41235-022-00354-0

**Published:** 2022-01-28

**Authors:** Ethan Kutlu, Mehrgol Tiv, Stefanie Wulff, Debra Titone

**Affiliations:** 1 Department of Psychological and Brain Sciences, University of Iowa, Iowa City, USA; 2 Department of Linguistics, University of Iowa, Iowa City, USA; 3 Department of Psychology, McGill University, Montreal, Canada; 4 Department of Linguistics, University of Florida, Gainesville, USA; 5 Department of Language and Culture, UiT The Arctic University of Norway, Tromsø, Norway

**Keywords:** Race, Audio-visual, Speech perception, Accents, Ecological diversity

## Abstract

Upon hearing someone’s speech, a listener can access information such as the speaker’s age, gender identity, socioeconomic status, and their linguistic background. However, an open question is whether living in different locales modulates how listeners use these factors to assess speakers’ speech. Here, an audio-visual test was used to measure whether listeners’ accentedness judgments and intelligibility (i.e., speech perception) can be modulated depending on racial information in faces that they see. American, British, and Indian English were used as three different English varieties of speech. These speech samples were presented with either a white female face or a South Asian female face. Two experiments were completed in two locales: Gainesville, Florida (USA) and Montreal, Quebec (Canada). Overall, Montreal listeners were more accurate in their transcription of sentences (i.e., intelligibility) compared to Gainesville listeners. Moreover, Gainesville listeners’ ability to transcribe the same spoken sentences decreased for all varieties when listening to speech paired with South Asian faces. However, seeing a white or a South Asian face did not impact speech intelligibility for the same spoken sentences for Montreal listeners. Finally, listeners’ accentedness judgments increased for American English and Indian English when the visual information changed from a white face to a South Asian face in Gainesville, but not in Montreal. These findings suggest that visual cues for race impact speech perception to a greater degree in locales with greater ecological diversity.

Despite its ubiquity in daily life, speech processing can be demanding (Brown et al., 2020). This demand is partly due to variability within- and between-speakers (Bradlow & Bent, 2008), and partly due to listeners’ finite cognitive resources (Pichora-Fuller et al., 2016) and their accumulated life experiences (Babel & Mellesmoen, 2019; Bradlow & Bent, 2008; Walker & Campbell-Kibler, 2015). One phenomenon that intersects with many of these factors is *non-native* accents^1^ (Bradlow & Bent, 2008; Porretta et al., 2016). Non-native accents (also see foreign-accented speech) are assumed to deviate from local accents (Cristia et al., 2012), and tend to be processed differentially by listeners (Floccia et al., 2009; Gass & Varonis, 1984; Mattys et al., 2012; Munro & Derwing, 1995a, 1995b; Van Engen & Peelle, 2014), although adaptation to accents depends on listeners’ exposure level (Baese-Berk et al., 2013; Bradlow & Bent, 2008; Brown et al., 2020; Clopper & Pisoni, 2004; Porretta et al., 2016).

Foreign accented speech, as distinguished from other variations of regional dialects, is often associated with multilingual speakers, and is assumed to have arisen from segmental (i.e., vowels or consonants) and suprasegmental (i.e., tone, intonation, stress) differences between a speaker’s first language (L1) and second language (L2) (Best et al., 2001; Flege, 1991; Guion et al., 2004; Trofimovich & Baker, 2006). Listeners can judge whether a speaker has an accent that is different than their own accent in milliseconds by tracking these segmental and suprasegmental differences (see Floccia et al., 2006, see review by Cristia et al., 2012).

This ability to detect accents develops during the first years of life. For instance, young infants prefer to look at individuals who share their *native* language (i.e., the language most spoken around the child) (Kinzler et al., 2007, 2010). Children as early as 5 years of age choose friends from various racial backgrounds who speak their *native* language (Kinzler et al., 2009). Importantly, children who live in multilingual communities exhibited biases against speakers who had accents different from their own (Byers-Heinlein et al., 2017; Paquette-Smith et al., 2019; Souza et al., 2013; but also see work that shows that bilingual children have less racial biases: Singh et al., 2019; Singh, Quinn, et al., 2020; Singh, Tan, et al., 2020). While these studies show that biases towards accented speech emerge early in childhood, it appears that the act of judging an accent is not merely developmental in nature; instead, linguistic, cognitive, and environmental factors shape accentedness judgments over a speaker’s lifespan.

The ability to perceive whether someone has a foreign accent goes beyond linguistic processes. Some have considered this ability to be an important part of social evolution in that accent detection contributes to humans’ “natural selection building system,” and is heuristically driven for instantaneous detection of in- versus out-group membership (Pietraszewski & Schwartz, 2014; see also Walker et al., 2018). Accents help us identify group relations (Dragojevic, 2020; Lippi-Green, 2012; Walker, 2010) in that hearing someone’s accent presents immediate socio-indexical information, such as race, ethnicity, place of birth, age, socioeconomic status, sexual orientation, and gender identity (Labov, 1986; Lippi-Green, 2012; McGowan, 2015; Munson & Babel, 2007; Pierrehumbert et al., 2004). These socio-indexical cues are important in everyday conversations for both speech perception (Sumner et al., 2014) as well as for a social understanding of certain facets of interlocuter identity. Given its association to multilingualism, here, we treat foreign-accented^2^ judgments as multilingual speech judgments, and examine how, through these judgments, multilinguals are implicitly subjected to foreignness or out-group membership.

The tendency to associate multilingualism with foreignness especially modulates those whose social status is already stigmatized. These stigmatizations further affect the ways in which multilinguals engage in conversation across professional, educational, and health settings, where someone with an out-group accent can be implicitly or explicitly treated differently due to their perceived accent (Itzhak et al., 2017; Kim et al., 2011). Therefore, a foreign accent that signals *out*-group membership has the potential to be associated with more negative attitudes (Giles & Watson, 2013; Gluszek & Dovidio, 2010a, 2010b; Lippi-Green, 1994, 2004), higher dysfluency in processing (Dragojevic, 2020), and a more strenuous listening effort (Van Engen & Peelle, 2014). Apart from this associated foreignness, perceiving an accent may stigmatize certain racial or ethnic groups (Kang & Rubin, 2009). Together, a growing body of convergent findings highlights the role played by foreign-accented speech in listeners’ speech perception. However, in real-world interactions, audio cues are processed alongside visual cues (e.g., the face of the speaker), and much less is known about the simultaneous audio-visual integration of accented speech (see Hansen et al., 2017; Paladino & Mazzurega, 2020).

Research on audio-visual language processing suggests that speech intelligibility (i.e., understanding the intended words) and accentedness judgments (i.e., the subjective evaluation of speech) can be modulated by seeing a face. Babel and Russell (2015) found that presenting an Asian face impeded listeners’ perception of Canadian English when compared to seeing a white face (see also Niedzielski, 1999). Similarly, Kutlu and colleagues (2020; 2021) showed that when presented with white faces American, British, and Indian English were found to be more intelligible and less accented. This was the opposite when the same recordings were presented with South Asian faces. Listeners were less accurate in their transcription when they saw a South Asian face on the screen, and they judged all English varieties as more accented. Crucially, they found that listeners who had more racial diversity in their social network judged all speech stimuli as less accented. On the other hand, McGowan (2015) found that listeners showed facilitation in their processing of Chinese-accented English when paired with Asian faces, suggesting that listeners associate race and ethnicity with accented speech.

Yi and colleagues (2013) merged their audio-visual experiment with an Implicit Association Test where they found that listeners who had greater associations between white faces and American places judged audio-visual trials with Korean faces as being more accented compared to audio-only trials. Zheng and Samuel (2017) also used videos to assess whether the mode of presenting visual information modulated speech perception. They found that listeners’ perception of speech was not affected by the videos but their accentedness judgments changed, which was not observed when presented with the static pictures. The neural correlates of integrated visual and linguistic processing have also been documented with event-related potentials (ERP). For instance, Grey et al. (2020) found that the P600 component, which indexes grammatical processing, was modulated depending on the race of the face cue, whereas the N400, which indexes semantic processing, was not modulated (see also Hanulíková and colleagues work on L1 and L2 speech: Hanulíková et al., 2012; Grey & van Hell, 2017). All these findings suggest that faces as well as racial information affect listeners’ judgments of the presented speech (see also Yi et al., 2014; Banks et al., 2015; Kutlu, 2020; Kutlu et al., 2021).

As Babel and Mellesmoen (2019) asserted, listeners’ experience with language and society builds their linguistic representations and, in turn, their expectations. Recent studies indicate that individuals who live in more diverse locales show different linguistic processing styles. For instance, Bice and Kroll (2019) trained monolingual participants to learn Finnish in two locales—one being linguistically diverse (California) and the other being less diverse (Pennsylvania). They found that in both locales, monolinguals demonstrated word learning in Finnish, but only in the linguistically diverse locale did they find neural evidence towards attendance to more subtle linguistic information measured by means of electroencephalography. While it is clear that living in linguistically diverse locales might allow learners to use subtle linguistic information more efficiently (see Tiv et al., accepted), others have also found that in lab training, monolingual listeners’ judgments can be reduced towards different foreign accents. For instance, Bradlow and Bent (2008) showed that hearing multiple speakers with Chinese-accented English helped listeners develop highly generalized cognitive representations of Chinese-accented speech. Baese-Berk and colleagues later found that exposing listeners to speakers from different language backgrounds during training helped them generalize their learning to novel speakers such that listeners were able to generalize their learning to both speakers that they were trained with during the training session as well as with speakers who were not in their training. They argued that generalizations of foreign accent adaptation are the result of exposure to systematic variability in accented speech (Baese-Berk et al., 2013). Nevertheless, it is unclear whether living in multilingual locales—where it is more common to be exposed to individuals who speak different languages or language varieties, and who belong to different racial and ethnic backgrounds—modulates listeners’ speech perception and modulates their accent perception. The novel contribution of the present study is to understand whether the impact of seeing static faces on speech processing depends on the diversity of one’s current community, and whether the linguistic ideologies of one’s community impact speech perception.

## The present study

This study investigates whether living in differing multilingual locales modulates speech perception and accentedness judgments towards three English varieties (i.e., American, British, and Indian English). These three varieties were chosen for several key reasons. Historically, these three varieties share the same linguistic past. Both American and Indian English emerged from British English, though one emerged as a result of British settlement (i.e., American English) while the other as a result of British colonialism (i.e., Indian English) (Kachru, 1986). Often, when compared to American and British English, Indian English is associated with lower prestige, more prejudice, and a higher degree of foreign accentedness (Kutlu, 2020; Kutlu & Wiltshire, 2020; Kutlu et al., 2021), despite their linked origins. Another difference that Indian English holds comes from its links to multilingual speakers. India houses over 120 mother tongues (Census of India, 2011), and as a result, Indian English speakers rarely speak solely Indian English. Due to its multilingual nature, Indian English is often perceived as a foreign accent compared to American and British English, which are perceived as different varieties of English (Kutlu & Wiltshire, 2020). This multilingual aspect of Indian English has become further embedded into the racialization of South Asian individuals (Kutlu, 2020; Ramjattan, 2019; also for raciolinguistic ideologies see Rosa, 2016). For instance, while there are South Asian speakers of all three varieties, there is often perceived foreign accentedness when they speak American and British English—as shown by studies comparing the perception of identical speech samples paired with both South Asian and white faces (Kutlu, 2020; Kutlu et al., 2021). It is therefore important to assess whether such racially driven assumptions of accents are comparably found in multilingual spaces or locations where listeners are more likely to hear South Asian individuals speaking.

Thus, to investigate whether living in multilingual, multicultural locales modulates speech perception and accentedness judgments towards racialized varieties, we conducted two experiments: one in Gainesville, Florida (USA), and one in Montreal, Quebec (Canada). These multilingual locations offer a unique way to test whether variability in language exposure (e.g., linguistic diversity, political approaches to multilingualism, language policies) modulates foreign accent judgments. Gainesville is a small college town in Florida hosting primarily English–Spanish bilinguals. Although there is a substantial amount of Spanish spoken in Florida, there are negative attitudes associated both with speaking Spanish (Kutlu & Kircher, 2021) as well as with speaking other varieties of English (Kutlu & Wiltshire, 2020). Importantly, these attitudes are shaped by monolingual ideologies in the U.S., reflected by prejudice towards being a bilingual in the U.S., as being bilingual/multilingual (and bi/multicultural) is often associated with being “un-American” (Kircher & Kutlu, under review). Therefore, bi/multilinguals in the U.S. do not have equitable access to their non-English languages (Devos & Banaji, 2005; Rosa, 2016).

Unlike Gainesville (U.S.), Montreal is an urban, multilingual city with a large English-French bilingual population (see e.g., Tiv et al., *accepted*; Tiv et al., 2020). In contrast to the U.S., where bilingualism is generally considered a deficit rather than a resource (Ricento, 2013), Canada is officially a bilingual country. Bilingualism is supported socioculturally in Montreal, although the use of French is legislated through language policy and planning measures in order to maintain the vitality of the language (e.g., Bill 101). Therefore, the two locales, despite both being home to multilinguals, differ in terms of their engagement with multilingualism. This unique difference between the two countries provides opportunities to test the potential impact of language ideologies on speech perception.

## Methods

### Participants

**Fig. 1:**
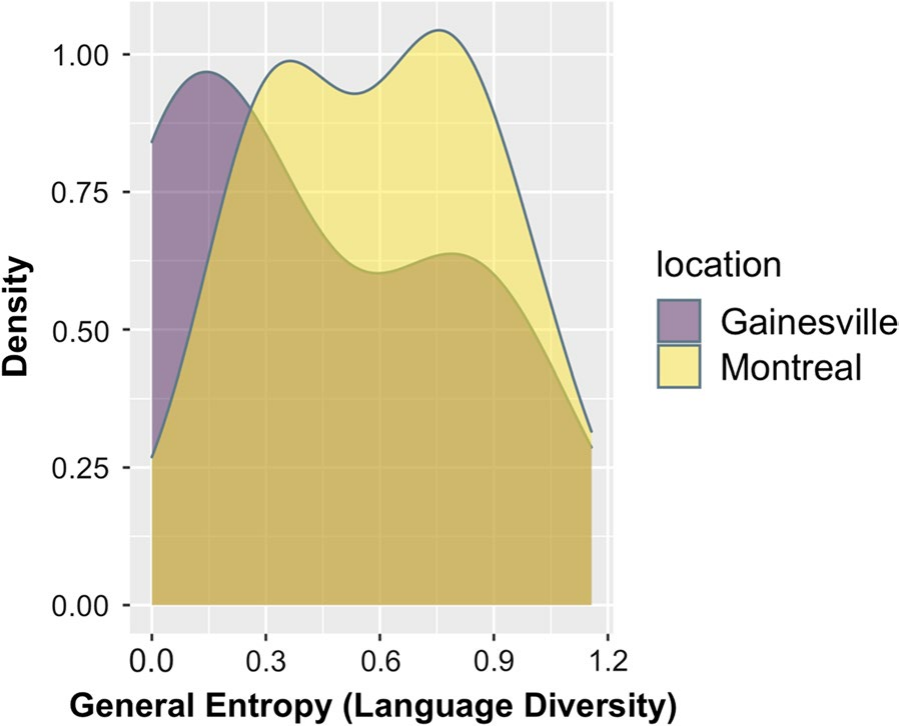
Overall language entropy distribution in Montreal and in Gainesville

Fifty participants were tested, 25 in each locale. (see Table 1). Participants were all undergraduate students in Gainesville and in Montreal. Since all participants were exposed to either Spanish or French to a certain degree and they self-identified as bilinguals, the LexTale English proficiency test was administered. The results of the proficiency test showed that participants in Montreal (*M* = 86.3) and Gainesville (87.4) did not statistically differ in their English proficiency. In Montreal, 7 out of 25 participants indicated that they were not born in Montreal. However, all 7 participants indicated that they had lived in Montreal for at least 1 year or more (Max year = 4 years, Min year = 1 year, Mean year = 2.4 years). In Gainesville, 5 participants indicated that they were born outside of Florida. These participants also indicated that they spent at least 1 year in Gainesville (Max year = 3 years, Min year = 1 year, Mean year = 2 years). Since participants live in different locales, we quantified the linguistic diversity that they encounter every day through language entropy (Gullifer & Titone, 2020). This was done following the languageEntropy package (Gullifer & Titone, 2020) on self-reported percent daily language use. For Montreal participants, mean entropy was 0.58, and for Gainesville participants, mean entropy was 0.42, meaning that Montreal participants were more integrated in their everyday language use of English and French compared to Gainesville participants in their use of English and Spanish use (see Fig. 1).^3^ Overall, two participants in Gainesville and one participant in Montreal were excluded due to not having fully completed the experiment or for technical issues.

**Table 1:** Descriptive demographic background information

	Sample (*N* = 50)			
	Gainesville		Montreal	
	*M (SD)*	*n*	*M (SD)*	*n*
Age	20.4 (1.2)	25	20.4 (1.5)	25
Age of Acquisition				
English	0.68 (1.4) Min = 0, Max = 4		0.04 (0.2) Min = 0, Max = 1	
French	0.56(2.8) Min = 14, Max = 14		3.08(2.1) Min = 0, Max = 5	
Spanish	4(6.1) Min = 0, Max = 14		3.52(5.8) Min = 11, Max = 15	
Daily use (%)				
English	87.6 (13.8)		84.48(13.3	
French	0 (0)		13.8(13.1)	
Spanish	11.6(13.6)		0.08(0.2)	
Gender				
Female		22		24
Male		2		1
Queer/Non-binary		1		
Racial/Ethnic Identity				
Black				
East Asian		1		2
Middle Eastern				1
Pacific Islander				
Southeast Asian		1		
White		12		19
Latin American		3		
Bi/multiracial		8		3

### Design

To measure whether listeners’ perception of a speaker’s accent changes when presented with a face, we used the same audio-visual experiment as Kutlu and colleagues used (Kutlu, 2020; Kutlu et al., 2021), where participants saw an image (a face) on the computer screen and heard a sentence immediately after having seen the image. We counterbalanced the stimuli such that participants could hear all three varieties from a white face or a South Asian face, allowing us to assess whether participants’ judgments towards these varieties change depending on the face that they see on the screen. Participants completed the intelligibility task in which they were asked to listen to the sentences while seeing a face on the screen and to transcribe them. Then, they were asked to complete the accentedness judgment task by listening to a subset of these sentences and judging whether the speaker had an accent or not in relation to their own perceived accent. In between the two intelligibility and accentedness experimental components, participants completed a language background questionnaire (Li et al., 2019) and the LexTale English proficiency test (Lemhöfer & Broersma, 2012).

### Stimuli

#### Auditory stimuli

One hundred and twenty short sentences used in past studies (Bradlow & Alexander, 2007; Kutlu, 2020; Kutlu et al., 2021; McGowan, 2015) were recorded (see “Appendix B”). These sentences were normed in previous speech perception studies and controlled for their word frequency. Half of the sentences were designed to be highly predictable and the other half had low predictability.

For American English, we used recordings of two female speakers taken from the OSCAAR speech corpus.^4^ Six Indian English speakers were recorded at the University of Florida. Since Indian English speakers were multilinguals, we only recorded those who spoke Tamil, Telugu or both along with Indian English, to account for phonological differences that can be observed in Indian English. Tamil and Telugu were chosen as they are among the most widely spoken languages in India (Census of India, 2011). All speakers reported that they acquired Indian English from birth, and they all completed English schools in India before their arrival to the US. They were all graduate students who arrived in the US one semester prior to the recording session. All Indian English speakers self-identified their accent as standard Indian English. For the British English recordings, 6 female speakers who were born and raised in Reading, UK, and who self-identified their accent as Standard British English were recorded. All twelve speakers were paid $10 for each recording session, and each session took less than 2 h. Participants first practiced the sentences by themselves and were then asked to read them out aloud in quiet rooms.

All recordings were normalized for their volume in PRAAT prior to this experiment (Boersma & Weenik, 2017). Sixteen separate University of Florida undergraduate student judges evaluated the recordings from all speakers (see “Appendix A”). All these judges (*M*_age_ = 19.4 years, 8 self-identified as women, 6 self-identified as men, 2 self-identified as gender queer) self-identified their speech as American English. The objective LexTale English proficiency test scores had a mean score of 86. All judges were exposed to Spanish to some degree. Additional language background questions yielded that none of the judges had extensive exposure to either British or Indian English. However, all judges indicated more familiarity (i.e., Yes/No familiarity question) with British English compared to Indian English. During the norming task, participants did not see any visual information on the screen. They were asked to transcribe what they were listening to (Babel & Russell, 2015), and were instructed to guess where the speaker might be from. Given that intelligibility and correct identification of origin have been shown to impact listeners’ attitudes towards speech (Derwing & Munro, 2009), auditory stimuli with at least 85% intelligibility and correct identification of origin were kept. Based on the norming data (“Appendix A“), six female speakers for the actual experiment (two female speakers for each variety) were selected. To make the task slightly challenging for participants, and to assess whether noise modulates the intelligibility of different varieties of Englishes (see work by Van Engen & Bradlow, 2007; Van Engen et al., 2014), a − 4 dB (signal to noise ratio) white noise was added to the recordings (McGowan, 2015). This way, the task mimicked real-world scenarios where there is often background noise during speech perception.

#### Visual stimuli

For visual stimuli, two previously normed and controlled face databases were used. South Asian faces were taken from the KKWETC face database (Satone, 2017), and white faces were taken from the Chicago face database (Ma et al., 2015). From each database, three female faces that were shown to display no emotional valence were picked. Moreover, there were no piercings or tattoos on the faces that might make them stand out when compared to other faces. Once selected, all images were converted to black and white scale via Adobe Photoshop, and contrast was normalized across all pictures to eliminate any low-level visual processing information (e.g., luminance) as well as to make pictures as similar as possible. Six white and South Asian faces (3:3 ratio) were matched with three different accents by way of a fully randomized Latin-square counterbalance distribution.

### Procedure

**Fig. 2:**
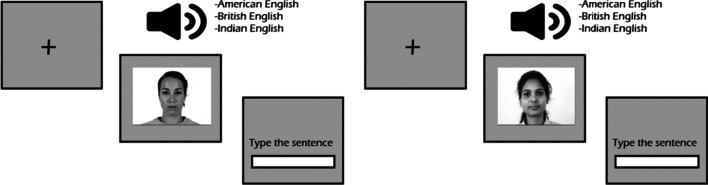
The design of the intelligibility task (both pictures are allowed to be used for publication purposes)

The intelligibility and accentedness judgment tasks were administered via PsychoPy (Peirce, 2007). Both tasks were always administered in the same order (i.e., intelligibility first). Two distinct scripts were created for each task, both of which are available on OSF (https://osf.io/9xgd8/?view_only=a68cac5b47464c5cbb2b9ba390ea0194). For the intelligibility task, participants were first shown an image on the computer screen. About 250 ms after the onset of the image, the auditory stimulus played. The image remained on the screen throughout the duration of time that participants were typing their sentences. This manipulation was done to prime participants with the socio-indexical information of the speaker (i.e., speakers’ race). Participants were asked to first listen to the short sentences and were then asked to start typing the sentences. Participants were instructed to ignore any punctuation and capitalization and were explicitly told to type as quickly and as accurately as possible (see Fig. 2). During the debriefing session, participants were asked if they had difficulty remembering the sentences while they were typing them, and no participant reported any difficulty or any other task-related issues.

**Fig. 3:**
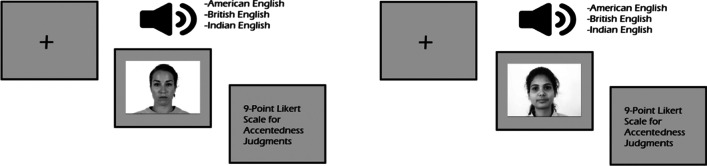
The design of the accentedness task

For the accentedness judgment task, participants were again prompted with an image that appeared 250 ms before the onset of the auditory stimuli. The image remained on the screen until participants judged the accentedness of the speech sample. Participants were told to wait until the end of the sentence and to rate the level of the accentedness on a 9-point Likert scale (with 1 being no accent and 9 being heavily accented^5^). Button-presses were also locked to avoid any early button-presses. Thus, participants were only able to press the button once the audio file played completely. Once the sentence ended and participants pressed a button, accentedness judgments were recorded (see Fig. 3). Participants were instructed regarding this information and completed a practice trial with the research assistant that consisted of three sentences, which were excluded from the analysis. Three practice trials were created randomly from all 6 speakers such that one speaker per variety was a possible practice trial to minimize familiarity towards a specific speaker and a specific variety. In the intelligibility task, participants listened to all 120 sentences. With a within-subject design, all three English varieties were presented with both a white face and a South Asian face during the experiment, and all items were counterbalanced such that no single sentence stimulus was presented with two different faces in the same list. As a result of these design considerations, there were two lists, and each participant was only tested with one of them.

For the accentedness judgments task, a subset of the same 120 sentences was used. To reduce the repetition effect, participants were asked to judge only a subset of the 120 sentences, yielding accentedness judgments for 60 sentences. The same face and speaker distribution from the intelligibility task were used. Since we used a subset of the 120 sentences from the intelligibility task, there were four counterbalanced lists (60 sentences from each of the two 120-sentence lists used in the intelligibility task), and each participant completed only one of the four lists.

Testing was completed in a quiet room. Participants were recruited through the SONA systems in both testing universities. The experiment took 1.5 h to complete, and participants received class credits upon their completion.

## Results

### Intelligibility scores

Intelligibility scores were operationally defined in terms of transcription accuracy (Porretta et al., 2016). For each sentence, content words were selected (see all capital letter words as an example of content words in a given sentence, e.g., a BOOK TELLS a STORY), and their transcription accuracy was calculated by means of 1s for correct content words and 0s for incorrect words. We chose to analyze all content words compared to the analysis of the final word as we wanted to measure whether participants’ engagement in the overall typing process from the beginning of a sentence (with high- or low-predictable sentences) would vary depending on the faces that they saw on the screen. Typos that were close to the target content words were counted as 1 (e.g., *tels* instead of *tells* or *bok* instead of *book*). Accuracy proportions were then calculated for each sentence (*n* = 120) and for each participant. To investigate accuracy proportions as a function of our independent variables—face, English variety, sentence predictability, and location—we constructed a linear mixed-effects model using the lme4 package (Bates et al., 2015) in R (R version 3.6.1; R Core Team, 2019). Follow-up tests were conducted with the lsmeans package (Lenth, 2017) and corrected pairwise comparisons with Bonferroni correction. Proportions were entered as the continuous dependent variable. As fixed effects (a) Helmert-coded Variety (American English vs. British English), and (Indian English vs. American + British English), (b) treatment coded Face (South Asian (1), white (2)), and (c) treatment coded Location (Gainesville (1), Montreal (2)). Treatment coded Predictability (High (1), Low (2)) was included as a covariate. Random effects were by-subject and by-item random intercepts and Predictability was added as a random slope to by-item. Other random slopes were eliminated from by-subject as the model did not converge. This model explained 24% of the variance in data and was the best fit when compared to other models.

Results showed that sentences paired with white faces were transcribed more accurately than those paired with South Asian faces (*b* = 0.07, *SE* = 0.007, *t* = 13.9, *p* < 0.001) (see Tables 2 and 3). Additionally, Indian English was transcribed less accurately compared to American and British English (*b* = − 0.008, *SE* = 0.004, *t* = − 1.67, *p* = *.* 0.09). No such difference was observed between American and British English (*b* = − 0.04, *SE* = 0.002, *t* = − 14.2, *p* < 0.001). Results also yielded significant differences between two locales such that Montreal participants were overall more accurate in their transcriptions compared to Gainesville participants (*b* = 0.06, *SE* = 0.01, *t* = 6.79, *p* < 0.001). These main effects were further qualified by multiple interactions.

**Table 2:** Summaries of the Mean and Standard Deviation (in parenthesis) of the accentedness judgments and intelligibility scores

	Gainesville		Montreal	
	White	South Asian	White	South Asian
*Accent Judgments*				
AmE	2.0(1.36)	3.5(1.90)	2.4(1.70)	2.2(1.63)
BrE	4.8(1.40)	5.6(1.80)	6.4(1.66)	6.5(1.5)
IndE	7.2(1.48)	7.5(1.38)	7.8(1.19)	7.6(1.06)
*Intelligibility*				
AmE	0.98(0.08)	0.97(0.09)	0.98(0.08)	0.98(0.07)
BrE	0.98(0.08)	0.93(0.16)	0.98(0.07)	0.98(0.10)
IndE	0.96(0.13)	0.83(0.28)	0.98(0.10)	0.97(0.11)

AmE refers to American English, BrE refers to British English, and IndE refers to Indian English

**Table 3:** Summary of the linear mixed-effects model results of the proportions as the dependent variable

	Proportion		
Fixed Effects	Estimates	SE	*P*-Value
Intercept	0.92	0.01	** < 0.001**
Face (white)	0.07	0.01	** < 0.001**
Variety (British vs. American)	− 0.01	0.00	0.094
Variety (Indian vs. American + British)	− 0.04	0.00	** < 0.001**
Location (Montreal)	0.06	0.01	** < 0.001**
Predictability(Low)	− 0.01	0.01	0.155
Face (white):Variety (British vs. American)	0.01	0.01	0.053
Face (white):Variety (Indian vs. American + British)	0.03	0.00	** < 0.001**
Face(white):Location(Montreal)	− 0.07	0.01	** < 0.001**
Variety (British vs. American):Location(Montreal)	0.01	0.01	0.305
Variety (Indian vs. American + British):Location(Montreal)	0.04	0.00	** < 0.001**
Face(white):Variety(British vs. American):Location(Montreal)	− 0.00	0.01	0.681
Face(white):Variety (Indian vs. American + British):Location(Montreal)	− 0.03	0.01	** < 0.001**
Observations	6120		
Marginal R^2^ / Conditional R^2^	0.092 / 0.232		

Significant results are in bold

First, there was an interaction between Face and English Variety (*b* = 0.04, *SE* = 0.005, *t* = 8.3, *p* < 0.001). Indian English paired with South Asian faces was transcribed less accurately compared to white faces (*b* = − 0.07, *SE* = 0.006, *t* = − 10.9, *p* < 0.001). Moreover, British English paired with South Asian faces was transcribed less accurately compared to white faces (*b* = − 0.03, *SE* = 0.006, *t* = − 5.4, *p* < 0.001). The third interaction was between Face and Location (*b* = − 0.065, *SE* = 0.007, *t* = − 8.53, *p* < 0.001) such that speech paired with South Asian faces was transcribed less accurately compared to white faces in Gainesville (*b* = − 0.07, *SE* = 0.006, *t* = − 13.9, *p* < 0.001), and speech paired with South Asian faces was transcribed less accurately in Gainesville compared to Montreal (*b* = − 0.06, *SE* = 0.009, *t* = − 6.79, *p* < 0.001). The third interaction was between Variety and Location (*b* = − 0.03, *SE* = 0.009, *t* = 9.81, *p* < 0.001). This interaction was driven by a more accurate transcription of American and British English compared to Indian English in Gainesville (*b* = 0.07, *SE* = 0.007, *t* = 10.05, *p* < 0.01; *b* = 0.07, *SE* = 0.007, *t* = 10.42, *p* < 0.01) and less accurate transcriptions of Indian English in Gainesville than in Montreal (*b* = − 0.08, *SE* = 0.01, *t* = − 7.44, *p* < 0.001).

**Fig. 4:**
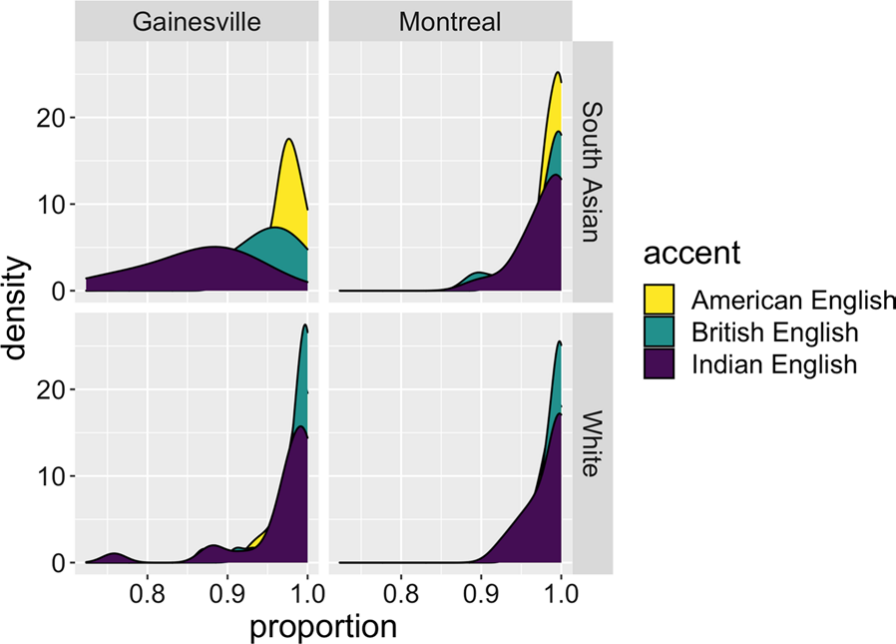
Proportions of intelligibility scores in Montreal and in Gainesville for each face and variety

Critically, there was a three-way interaction between Face * Variety * Location (*b* = − 0.03, *SE* = 0.005, *t* = − 5.5, *p* < 0.001). This interaction revealed that Indian English presented with South Asian faces was transcribed less accurately in Gainesville than in Montreal (*b* = − 0.14, *SE* = 0.01, *t* = − 11.4, *p* < 0.001) (Fig. 4).

**Fig. 5:**
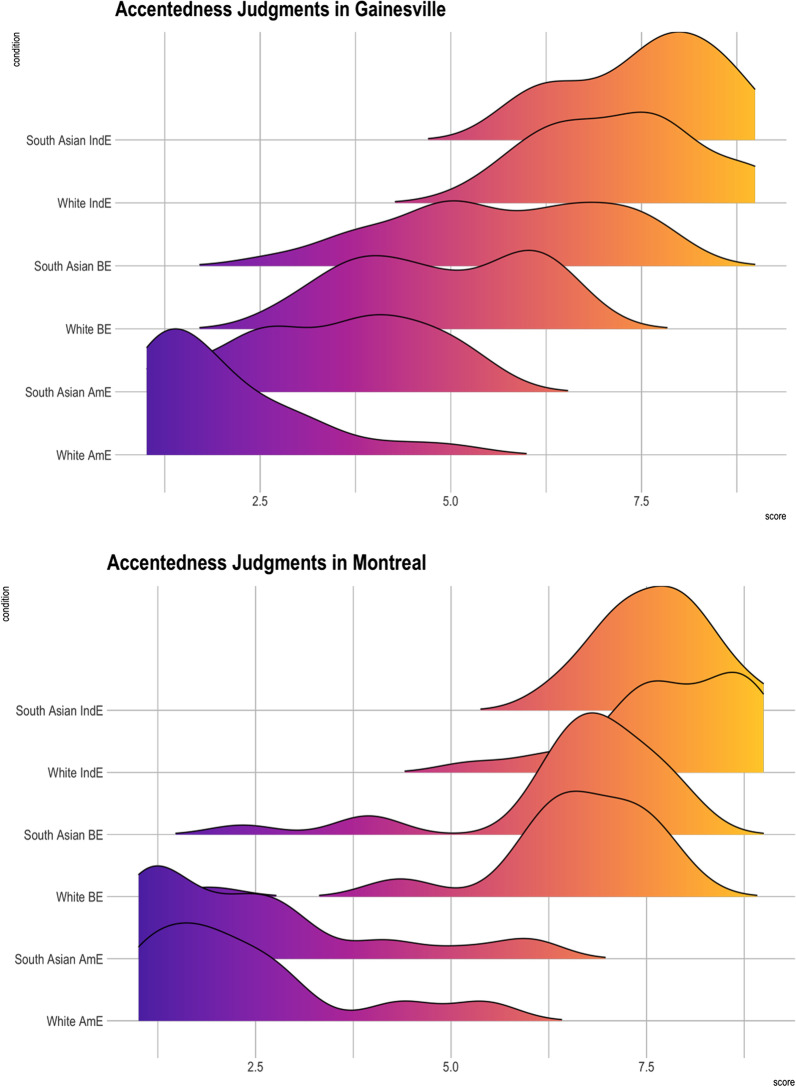
Accentedness judgments in Montreal and in Gainesville for each face and variety type

### Accentedness judgments

The accentedness scores varied from 1–9 on a 9-point Likert scale. We, therefore, treated the scores as ordinal data and used Cumulative Link Mixed Models (Christensen, 2015) which treats ordinal data as categorical. This analysis was completed through the ordinal package in R which comes with the cumulative link model for ordinal regression. Accent scores were the dependent variable and Variety (American, British, Indian English), Face (South Asian, white), and Location (Gainesville, Montreal) were entered as the fixed effects with the same contrast coding as the intelligibility analysis. Predictability (High, Low) was entered as a covariate. Random intercepts were by-subject and by-item. Random slopes were eliminated as the model did not converge. The model explained 72% of the variability in the data (Table 4).

**Table 4:** Summary of the Cumulative Link Mixed Model with accentedness judgments as to the dependent variable

	Proportion		
Fixed effects	Estimates	SE	*P*-Value
Face (white)	− 1.16	0.09	**< 0.001**
Variety (British vs. American)	1.30	0.08	**< 0.001**
Variety (Indian vs. American + British)	1.31	0.05	**< 0.001**
Location (Montreal)	− 0.10	0.29	0.716
Predictability (Low)	− 0.16	0.06	**0.01**
Face (white):Variety (British vs. American)	0.41	0.11	**< 0.001**
Face (white):Variety (Indian vs. American + British)	0.32	0.06	**< 0.001**
Face(white):Location(Montreal)	1.35	0.13	**< 0.001**
Variety (British vs. American):Location (Montreal)	1.27	0.12	**< 0.001**
Variety (Indian vs. American + British):Location(Montreal)	0.01	0.06	0.793
Face(white):Variety(British vs. American):Location(Montreal)	− 0.54	0.16	**< .01**
Face(white):Variety (Indian vs. American + British):Location(Montreal)	− 0.12	0.09	0.206
Observations	3060		
Marginal R^2^ / Conditional R^2^	0.635/0.721		

Significant results are in bold

Results showed that listeners judged white faces as less accented compared to South Asian faces (*b* = − 1.16, *SE* = 0.09, *z* = − 11.9, *p* < 0.001). Moreover, British English was judged as more accented compared to American English (*b* = 1.30, *SE* = 0.08, *z* = 15.5, *p* < 0.001), and Indian English was judged as more accented than both American and British English combined (*b* = 1.31, *SE* = 0.05, *z* = 24.7, *p* < 0.001). These main effects were also further qualified by multiple interactions.

There was an interaction between Face (white) and Variety type (British vs. American) (*b* = 0.41, *SE* = 0.11, *z* = 3.54, *p* < 0.001) and between Face (white) and Variety type (Indian vs. American + British) (*b* = 0.32, *SE* = 0.06, *z* = 4.76, *p* < 0.001). These interactions suggest that for both American and British English, whenever they were paired with white faces, these recordings were judged as less accented compared to when paired with South Asian faces. This effect was not observed for Indian English (*p* > 0.05).

There was another interaction between Face (white) and Location (Montreal) which shows that white faces were judged as more accented in Montreal than in Gainesville (*b* = 1.244, *SE* = 0.29, *z* = − 4.23, *p* < 0.001), while no such difference was observed for South Asian faces (*p* > 0.05). Location also interacted with Variety type (*b* = 1.27, *SE* = 0.12, *z* = 10.6, *p* < 0.001) such that British English was judged as less accented in Gainesville compared to Montreal (*b* = − 1.61, *SE* = 0.30, *z* = − 5.36, *p* < 0.001). No such difference was observed for American or Indian English.

Finally, there was a critical three-way interaction between Face, Variety type, and Location for the American vs. British accent contrast (*b* = − 0.54, *SE* = 0.16, *z* = − 3.26, *p* < 0.01). This interaction was driven by higher accentedness judgments towards American English paired South Asian faces in Gainesville when compared to Montreal participants (*b* = 1.39, SE = 0.32, *z* = 4.30, *p* < 0.001) as well as British English recordings being judged as more accented in Montreal compared to Gainesville when paired with both white and South Asian faces (*b* = − 2.07, SE = 0.32, *z* = − 6.46, *p* < 0.001; *b* = − 1.15, SE = 0.32, *z* = − 3.57, *p* = 0.02, respectively) (see Fig. 5).

## Discussion

This study investigated how living in different multilingual locales modulates speech perception and accentedness judgments of three English varieties (i.e., American, British, and Indian English) when presented with white and South Asian faces. To test this, we conducted two experiments in two different locales, Gainesville and Montreal, that differed in terms of their multilingualism and multiculturalism. Overall, our findings showed that living in a locale where multilingualism is not promoted modulated speech perception, particularly when it was paired with South Asian faces. We discuss these findings in detail below.

First, we found locale differences such that Montreal participants were overall more accurate in their transcriptions compared to Gainesville participants. This difference could arise from the linguistic uncertainty that Montreal and Gainesville participants have in their everyday life. A recent framework proposed by Gullifer and Titone (2021) argues that there is individual variability in how bilinguals experience language-related uncertainties (see also Beatty-Martinez & Titone, 2021, who advocate for characterizing bilinguals through behavioral phenotyping). Language entropy is one way of measuring such fluctuations in different contexts. Here, we observed that bilinguals in Montreal and in Gainesville differed in terms of their overall language entropy. While Gainesville participants were in a more monolingual-like language state (i.e., low language entropy), Montreal participants were more bilingual in their everyday life (i.e., high language entropy). This suggests that Montreal participants are potentially more prone to experiencing linguistic uncertainties, which can then lead them to engage cognitive processes that handle such differences. On the other hand, it may not be cognitively adaptive for Gainesville bilinguals to engage in such processes as they do not encounter the same levels of language-based uncertainties in their everyday life. These findings also support the hypothesis that systematic exposure to variability aids speech perception (Baese-Berk et al., 2013). The observed differences between Montreal and Gainesville are important to discuss in speech perception research as these findings suggest a more context-based social information processing (see Hanulíková et al., 2012). Recall that all speech recordings were previously normed without any visual information to have at least 85% intelligibility. Our findings also show that these highly intelligible recordings became less intelligible when they were paired with South Asian faces compared to white faces, consistent with past work on how race modulates speech perception (Babel & Russell, 2015; Rubin, 1992; Hanulíková et al., 2012).

In terms of accentedness judgments, Montreal participants did not judge speech paired with South Asian faces as more accented compared to white faces within the same variety. For instance, there was no difference between white and South Asian faces for American, British, or Indian English. However, in Gainesville, both American and British English were judged as more accented when paired with South Asian faces. These differential mechanisms that are engaged for accentedness judgments in Gainesville and Montreal suggest that the social meaning of observable race has different values in these two locales. For instance, race is a less reliable cue in Montreal compared to Florida as it was seen that Montreal participants primarily use speech variables to assess one’s accentedness level, while Gainesville participants use both face and accents to make their judgments (also see Hanulíková et al., 2012). It is also crucial to note that overall, we replicated both our intelligibility and accentedness judgments findings in the previous study which was only conducted in Gainesville, Florida with a larger sample size (Kutlu et al., 2021). However, we found differences across different locales suggesting that context modulates the intelligibility and the accentedness judgments of speech.

Importantly, we found differences in accentedness judgments in terms of which variety the participants were listening to. British English was judged as less accented in Gainesville compared to Montreal. The higher accentedness judgments towards British English in Montreal suggest that for Montreal listeners, British English was not closer to their own variety type, but it was as accented as Indian English. This is an important finding. As we discussed earlier, Indian English speakers are those who speak many languages along with Indian English. These Indian English speakers grow up speaking Indian English as their own variety of speech. Therefore, perceiving Indian English as a more foreign variety suggests that listeners (i.e., Western listeners) associate Indian English with out-group members of the English-speaking community (Kachru, 1986; Kutlu, 2020). However, it seems like listeners in the different locales have different ways of positioning Indian English and British English speakers. While in Montreal, both British and Indian English are categorized towards the foreign variety, in Gainesville, British English serves as the intermediate step towards foreignness. This suggests that British English is not perceived as foreign as Indian English is to listeners.

We acknowledge several limitations of our design. We chose the sentences as they have been widely used in previous speech perception research. However, the sentence list consists of half high-predictable (i.e., *The color of a lemon is yellow*) and half low-predictable sentences (i.e., *The towel is yellow*). For the intelligibility task, it would be ideal to have all low-predictable sentences. Importantly, we normed all recordings without any visual information to have at least 85% intelligibility. This biases speech recordings to be highly intelligible. However, everyday interactions do also contain unintelligible speech. Findings in future studies might differ depending on the norming process. Nonetheless, these experimental contradictions should be interpreted as the need for speech perception research that is geared towards understanding social information processing (Hanulíková, 2021).

Our results here reinforce the role of race in speech perception. Further, they speak to how multilingual environments, race, and speech perception are intertwined. More studies are needed to understand the connection between foreign accents and race and how listeners form their associations towards multilingual groups and the ways in which they converge or diverge with multilingual speakers (Walker & Campbell-Kibler, 2015). We encourage researchers across the language and cognitive sciences to continue pursuing these important questions, and to acknowledge the diverse multilingual experiences and how these experiences shape their cognitive, emotional, and linguistic development (see e.g., Tiv, Kutlu, & Titone, 2020; López, 2020).

## Data Availability

Experimental coding scripts are available via the Open Science Framework under https://osf.io/9xgd8/?view_only=a68cac5b47464c5cbb2b9ba390ea0194. The datasets analyzed during the current study are available from the corresponding author on reasonable request.

## Appendix A

### Norming study

To assess that all Englishes have the similar comprehensibility, we measured the intelligibility of each speaker with a full transcription task both with and without noise. Intelligibility scores were analyzed in two ways: (i)scoring the accuracy of content words (e.g., MOM READ about the COLORS), (ii) scoring the final word accuracy. Recall that all sentences were from a previously tested and normed study (Bradlow & Alexander, 2007) with half of them being highly predictable and the other half being low predictable. In all sentences, the target word was at the end of the sentence (e.g., HP: The color of a lemon is **yellow**; Mom thinks that it is **yellow**). Since some of the sentences were not fully transcribed (i.e., missing final words), we chose the content word analysis over the final word analysis. Therefore, the analysis of content word percentages and proportions were used in the table below to show the differences among speakers.

A Python script was used to record intelligibility data. Following Babel and Russell (2015), we did not use any visual information during the intelligibility norming task to avoid any social cues. This was done to maximize the acoustic characteristics of the speech since our goal was to have recordings that have a similar intelligibility range. This similarity would allow us to assess whether participants use social cues such as the race to inform their accentedness judgments. Participants (*n* = 16, 9 female, M_age_ = 19.1) heard 120 sentences and then were asked to transcribe as accurately as possible. They were told to ignore any punctuation and were instructed not to use capital letters. At the end of the experiment, judges were asked if they could identify the accent of the speakers and in all cases, accents were identified correctly as American English, British English, and Indian English for all speakers. This served as the correct identification of speakers’ origin. Another set of participants were recruited to test the intelligibility in noise (*n* = 10, 7 female, *M*_age_ = 19.5). All participants reported speaking only American English and did not report extensive familiarity with Indian English (measured via the same Language Background Questionnaire that participants filled out). About 70% of the participants reported knowledge of Spanish from schooling. To avoid any repetition effect from typing sentences, there were 4 blocks. During these blocks, participants filled out language questionnaires and also completed the same LexTale English proficiency that was used in the actual experiment (*M*_score_ = 85).

To process the transcriptions, each participants’ transcriptions were entered into an Excel file and then was split into words using Microsoft Excel’s text-to-columns feature. Two research assistants went through each word per sentence and marked words that were not typed correctly with 0. All words that were typed correctly had 1. Typographical errors (e.g., “aple” for “apple”) were not counted as conceptual errors, and therefore, were accepted as correct. Furthermore, judges did not have issues typing high and low predictable sentences as there was no effect of predictability on intelligibility scores (*p* > 0.05). Therefore, percentages and proportions were created for the content word correctness. Table 5 shows the percentages of intelligibility for each speaker with and without noise. Two female speakers of British English and Indian English were picked based on their 85% above intelligibility (Table 5).

**Table 5:** Percentage of intelligibility with(n = 10) and without noise (n = 14) for each American English, British English and Indian English speaker. Only speakers who had 85% and above intelligibility were used in the actual experiment indicated with *

	English	Other language(s)	Percentage of intelligibility	Percentage of intelligibility with noise
Speaker 1*	American	None	98%	92%
Speaker 2*	American	None	98.5%	90%
Speaker 3*	British	None	96.2%	90%
Speaker 4	British	None	82%	78%
Speaker 5	British	None	92%	83.4%
Speaker 6	British	None	90%	82%
Speaker 7	British	None	93%	84%
Speaker 8*	British	None	94.4%	88%
Speaker 9	Indian	Tamil	77%	65%
Speaker 10	Indian	Tamil	81%	79%
Speaker 11*	Indian	Tamil, Telugu	91%	89%
Speaker 12	Indian	Tamil	68%	65%
Speaker 13*	Indian	Telugu	87%	85%
Speaker 14	Indian	Telugu	79%	72.5%

## Appendix B

### High predictable sentences

1. The meat from a pig is called pork.

2. For dessert he had apple pie.

3. Sugar tastes very sweet.

4. The color of a lemon is yellow.

5. My clock was wrong, so I got to school late.

6. In spring, the plants are full of green leaves.

7. A bicycle has two wheels.

8. She made the bed with clean sheets.

9. The sport shirt has short sleeves.

10. He washed his hands with soap and water.

11. The child dropped the dish and it broke.

12. The bread was made from whole wheat.

13. The opposite of hot is cold.

14. A wristwatch is used to tell the time.

15. The warplane dropped a bomb.

16. She cut the cake with a knife.

17. A chair has four legs.

18. Cut the meat into small pieces.

19. The team was trained by their coach.

20. The lady wears earrings in her ears.

21. People wear shoes on their feet.

22. When sheep graze in a field, they eat grass.

23. A rose is a type of flower.

24. Football is a dangerous sport.

25. The heavy rains caused a flood.

26. Bob wore a watch on his wrist.

27. Monday is the first day of the week.

28. The pan that was just in the oven is very hot.

29. Rain falls from clouds in the sky.

30. The boy laughed because the joke was very funny.

31. To cool her drink, she added a few cubes of ice.

32. A quarter is worth twenty-five cents.

33. An orange is a type of fruit.

34. People wear scarves around their necks.

35. I wrote my name on a piece of paper.

36. For your birthday I baked a cake.

37. Birds build their nests in trees.

38. My parents, sister and I are a family.

39. The good boy is helping his mother and father.

40. People wear gloves on their hands.

41. A book tells a story.

42. A pigeon is a kind of bird.

43. The sick woman went to see a doctor.

44. The lady uses a hairbrush to brush her hair.

45. At breakfast he drank some orange juice.

46. Last night, they had beef for dinner.

47. A race car can go very fast.

48. Many people like to start the day with a cup of coffee.

49. He brought the book to school from home.

50. I wear my hat on my head.

51. Red and green are colors.

52. The stars come out at night.

53. February has twenty-eight days.

54. The picture is hung high on the bedroom wall.

55. We heard the ticking of the clock.

56. She laid the meal on the table.

57. She looked at herself in her mirror.

58. Elephants are big animals.

59. After my bath, I dried off with a towel.

60. In the morning it gets light, and in the evening it gets dark.

### Low predictable sentences

1. Dad looked at the pork.

2. Mom talked about the pie.

3. We think that it is sweet.

4. Mom thinks that it is yellow.

5. He thinks that it is late.

6. She talked about the leaves.

7. He read about the wheels.

8. Dad talked about the sheets.

9. He looked at the sleeves.

10. We talked about the water.

11. We heard that it broke.

12. Dad pointed at the wheat.

13. She thinks that it is cold.

14. This is her favorite time.

15. Dad talked about the bomb.

16. Mom read about the knife.

17. She looked at her legs.

18. There are many pieces.

19. We read about the coach.

20. She pointed at his ears.

21. Mom looked at her feet.

22. Dad pointed at the grass.

23. She read about the flower.

24. This is her favorite sport.

25. He read about the flood.

26. He looked at her wrist.

27. This is her favorite week.

28. Mom thinks that it is hot.

29. Dad read about the sky.

30. Dad thinks that it is funny.

31. He talked about the ice.

32. He pointed at the cents.

33. He pointed at the fruit.

34. She talked about their necks.

35. We talked about the paper.

36. This is her favorite cake.

37. He read about the trees.

38. We read about the family.

39. Mom pointed at his father.

40. She looked at her hands.

41. We looked at the story.

42. We pointed at the bird.

43. Mom talked about the doctor.

44. He pointed at his hair.

45. Mom looked at the juice.

46. He talked about the dinner.

47. She thinks that it is fast.

48. Mom pointed at the coffee.

49. She pointed at the home.

50. She pointed at her head.

51. Mom read about the colors.

52. This is her favorite night.

53. There are many days.

54. We pointed at the wall.

55. She looked at the clock.

56. Dad read about the table.

57. We looked at the mirror.

58. He pointed at the animals.

59. Dad looked at the towel.

60. Dad thinks that it is dark.

## Footnotes

1 It should be noted that the term *non-native* and *native,* for that matter, are problematic and are found to racialize speakers of multilingual and/or different varieties. Here, we use this term only to refer to previous studies (for a detail discussion, see Baese-Berk et al., 2020; Cheng et al., 2021; Dewaele, 2018).

2 It should be noted that a foreign accent can be associated with a regional variety as well. However, regional differences are outside the scope of this study.

3 Integrated refers to the use of both languages in a more balanced way while compartmentalized refers to the single language use.

4 (OSCAAR: The Online Speech/Corpora Archive and Analysis Resource, talker speakers 439 and 441 (filenames: SC_S_ENF_29_EN, SC_S_ENF_18_EN).

5 No accent and heavily accented terms were used in relation to listeners’ own perceived accent. All listeners were instructed to listen to whether the speaker’s accent differed from their own accent.
